# Functional analysis and comparative genomics of expressed sequence tags from the lycophyte *Selaginella moellendorffii*

**DOI:** 10.1186/1471-2164-6-85

**Published:** 2005-06-06

**Authors:** Jing-Ke Weng, Milos Tanurdzic, Clint Chapple

**Affiliations:** 1 Department of Biochemistry, Purdue University, West Lafayette, IN 47907, USA; 2 Department of Botany and Plant Pathology, Purdue University, West Lafayette, IN 47907, USA; 3 current address, Cold Spring Harbor Laboratory, Cold Spring Harbor, NY 11724, USA

## Abstract

**Background:**

The lycophyte *Selaginella moellendorffii *is a member of one of the oldest lineages of vascular plants on Earth. Fossil records show that the lycophyte clade arose 400 million years ago, 150–200 million years earlier than angiosperms, a group of plants that includes the well-studied flowering plant *Arabidopsis thaliana*. *S. moellendorffii *has a genome size of approximately 100 Mbp, as small or smaller than that of *A. thaliana*. *S. moellendorffii *has the potential to provide significant comparative information to better understand the evolution of vascular plants.

**Results:**

We sequenced 2181 Expressed Sequence Tags (ESTs) from a *S. moellendorffii *cDNA library. One thousand three hundred and one non-redundant sequences were assembled, containing 291 contigs and 1010 singletons. Approximately 75% of the ESTs matched proteins in the non-redundant protein database. Among 1301 clusters, 343 were categorized according to Gene Ontology (GO) hierarchy and were compared to the GO mapping of *A. thaliana *tentative consensus sequences. We compared *S. moellendorffii *ESTs to the *A. thaliana *and *Physcomitrella patens *EST databases, using the tBLASTX algorithm. Approximately 60% of the ESTs exhibited similarity with both *A. thaliana *and *P. patens *ESTs; whereas, 13% and 1% of the ESTs had exclusive similarity with *A. thaliana *and *P. patens *ESTs, respectively. A substantial proportion of the ESTs (26%) had no match with *A. thaliana *or *P. patens *ESTs.

**Conclusion:**

We discovered 1301 putative unigenes in *S. moellendorffii*. These results give an initial insight into its transcriptome that will aid in the study of the *S. moellendorffii *genome in the near future.

## Background

Our understanding of biology has been greatly improved by studying genome structure and gene function of a broad sampling of model organisms such as *Mus musculus *(mouse), *Drosophila melanogaster *(fruit fly), *Danio rerio *(zebrafish), *Caenorhabditis elegans *(nematode), and *Arabidopsis thaliana *[1-5]. Comparative genomics has made it clear that orthologs of many proteins that act as signal transduction components, transcriptional regulatory factors, and metabolic enzymes can be identified between and among these model organisms [6]. As a result, the knowledge gained from comparative and evolutionary studies of these species can provide insights into homologous processes in a wide range of other organisms, varying from crop plants to humans [7]. Within plants however, most of the efforts in genomics have been focused on crop plants or economically important plants such as *Oryza sativa *(rice), *Zea mays *(maize), and *Lycopersicon esculentum *(tomato) [8-10]. Thus, coupled with the sequencing of the *A. thaliana *genome, these efforts have provided data on only a single branch of the plant evolutionary tree, namely members of the *Monocotyledonae *and *Dicotyledonae*, collectively termed the angiosperms and commonly known as flowering plants. As a result, the community of plant scientists has little sequence data on other plant lineages that could provide insights into common mechanisms of how plants develop and survive in a terrestrial environment, nor do they have any kind of evolutionary benchmarks that might reveal how angiosperms have come to dominate most world ecosystems [11].

**Figure 1:**
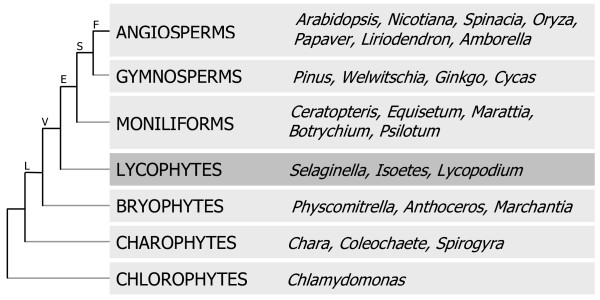
**A simplified version of the plant phylogenetic tree simplified and condensed from Pryer et al. [11]. **The tree shows that lycophytes (highlighted) diverged from other vascular plant lineages soon after plants colonized the terrestrial environment. Representative species were chosen from sub-clades within the clades listed, and illustrate major developments in plant evolution including the colonization of land (land plants, L), the development of vasculature (vascular plants, V) and true leaves (euphyllophytes, E), and the evolution of flowers (flowering plants, F), and seeds (seed plants, S).

Clear evidence for the existence of angiosperms is present in the fossil record of the lower Cretaceous (140 million years ago), and some evidence suggests their existence 60 million years earlier, around the same time that conifers and ginkgos arose [12]. In contrast, fossil evidence for the lycophytes is found in strata dated to approximately 420 million years ago [13]. Thus, this clade diverged very early from the lineage that led to all other vascular plants (Figure 1), and has existed on earth over twice as long as plants that are the most common subjects of current laboratory and agricultural research. As such, the study of lycophytes may provide novel insights into plant biology that would not be provided by research that focuses only on flowering plants.

**Figure 2:**
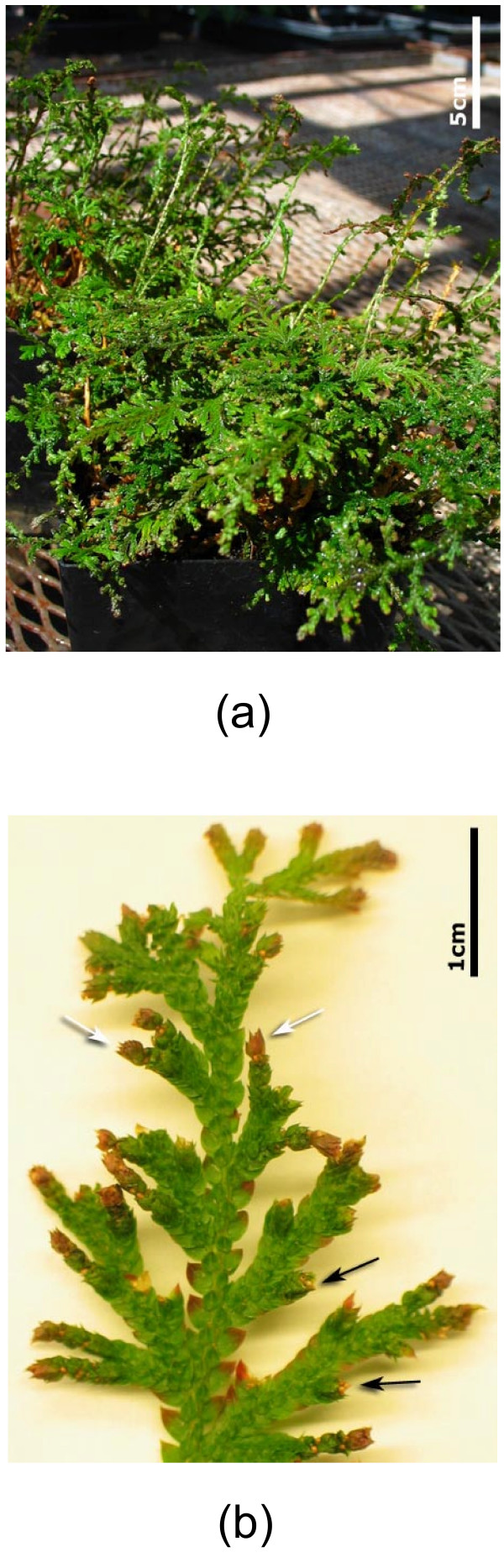
**The morphology of *S. moellendorffii*. **(a) A greenhouse grown *S. moellendorffii*. (b) A close up of an aerial branch of *S. moellendorffii *indicating the bulbils (white arrows) that can be used for clonal propagation and sporangia (black arrows) containing microspores and megaspores for sexual propagation.

*Selaginella *is an extant genus of the lycophyte clade. It is sometimes referred to as a 'seed-free' plant to highlight the fact that it has not evolved flowers and seeds in the time since its divergence from other plant lineages. It has a number of characteristics that would make its study convenient for, and valuable to, the plant biology community [11,14]. For example, like many other species of *Selaginella*, *S. moellendorffii *(Figure 2) is a small diploid plant that can be easily grown in the laboratory. Further, it has an approximate genome size of 100 Mbp [14], smaller than that of *A. thaliana*, and among the smallest published genome sizes for 'seed-free' genera. Because of these attributes, *S. moellendorffii *was recently chosen as one of the non-crop plants for BAC library construction in a NSF funded Green Plant BAC library Project [15]. More importantly, the Department of Energy Joint Genome Institute (JGI) has officially announced that it will sequence the *S. moellendorffii *genome [16], making this species a target of extreme interest for research into comparative plant genomics, biochemistry, and development.

Expressed sequence tag (EST) sequencing has been used as an efficient and economical approach for large-scale gene discovery [17]. It has also successfully provided frameworks for many genome projects [18,19]. Recently, a large number of ESTs have been generated from various plant species and deposited in GenBank, including both model and crop plants like *A. thaliana*, rice, wheat, and maize as well as species representative of clades other than angiosperms, such as gymnosperms, cycads, and mosses [20-23]. Although over 1000 ESTs from another *Selaginella *species *S. lepidophylla*, also known as the resurrection plant, have also been deposited in GenBank [20], no manuscript has been published reporting on their analysis. In this paper, we describe 2181 ESTs generated from a *S. moellendorffii *cDNA library. These ESTs were assembled into 1301 clusters, annotated using the BLASTX algorithm, surveyed for their abundance within the dataset, and classified into functional groups according to the Gene Ontology (GO) hierarchy. Finally, a comparative genomics approach was used for comparing *S. moellendorffii *ESTs with those of *A. thaliana *and *Physcomitrella patens *to look for genes unique to *S. moellendorffii*.

## Results and Discussion

### Generation of *S. moellendorffii *cDNA library and ESTs

To gain a broad coverage of *S. moellendorffii *transcripts, we collected and pooled whole *S. moellendorffii *plants for mRNA extraction and subsequent cDNA library construction. To enrich for full-length cDNA clones, double-stranded cDNA was size-fractionated before cloning. Based upon the average insert sizes of 35 cDNA clones chosen at random from the library, we estimate that the cDNA library has an average insert size of 850 bp. 2304 clones were sequenced from the 5' end of the cDNAs, which generated 2181 vector-trimmed EST sequences with an average sequencing read length of 640 bp.

### Assembly of *S. moellendorffii *ESTs

**Figure 3:**
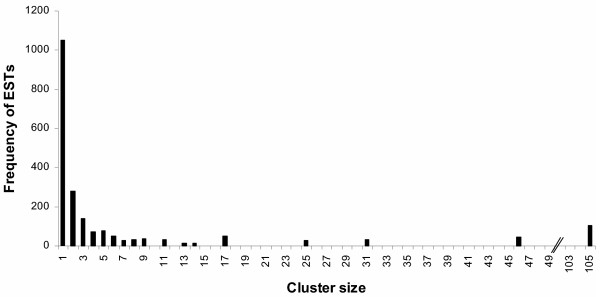
**Distribution of *S. moellendorffii *ESTs by cluster size. **ESTs were clustered into putative unigene sets using StackPack v. 2.2, and the number of cluster members of each size category was plotted relative to their abundance within the EST collection.

To identify overlapping EST sequences, reduce sequencing error and produce non-redundant EST data for further functional annotation and comparative analysis, 2181 ESTs were assembled into clusters through stackPACK v2.2 clustering system [24]. Based upon regions of nucleotide identity, EST sequences were merged into contiguous consensus sequences (contigs). One thousand three hundred and one non-redundant EST clusters, putatively regarded as unigenes, were generated, consisting of 291 contigs and 1010 singletons. The cluster size varied from one to 105 copies of any given EST (Figure 3). Manual inspection of the assembled ESTs identified 10 clusters counted as unigenes that may actually represent non-overlapping sequence reads from cDNAs corresponding to four single genes. As an example, three unigenes were found to be best aligned to three different regions of the same protein in a BLASTX analysis (described in the following paragraph), suggesting we lack a complete transcript for their accurate assembly. Conversely, we also found that some clustered ESTs did not necessarily have identical sequences within their overlapping regions. In most of the cases, regions of sequence disagreement within the clusters tend to appear towards the ends of the EST reads, which is likely to be caused by errors generated during sequencing. In some other cases, it may due to failure to discriminate between gene family members during clustering, or allelic diversity in *S. moellendorffii*.

### Annotation of *S. moellendorffii *ESTs

To annotate *S. moellendorffii *ESTs, the 1301 putative unigenes were translated dynamically in all 6 reading frames and searched for homology against the NCBI non-redundant (nr) protein database using BLASTX [25]. BLASTX hits with E-values less than 10^-5 ^were taken to be significant. Among 1301 unigenes, 962 (74%) had BLASTX hits in the nr database, while the remaining 339 (26%) had hits with E-values greater than 10^-5 ^or no hit. When a less permissive cutoff E-value of 10^-10 ^was adopted, the numbers of unigenes with BLASTX hits and without BLASTX hits changed slightly to 891 (68%) and 410 (32%) respectively. Our dataset showed that the inferred translation products of most *S. moellendorffii *ESTs appear to be similar to proteins in other organisms but that there was also a percentage of ESTs that represented potential *Selaginella- *or lycophyte-specific genes. Interestingly, 15 ESTs had at least their top five BLASTX hits from non-plant organisms, including six from bacteria or cyanobacteria (SmoC-1_02_N06, SmoC-1_01_C17, SmoC-1_02_B19, SmoC-1_06_K12, SmoC-1_cn167, SmoC-1_03_D21), two from fungi (SmoC-1_06_O23, SmoC-1_02_H20), one from an insect (SmoC-1_06_K02), three from nematodes (SmoC-1_04_D10, SmoC-1_02_L08, SmoC-1_cn108), one from fish (SmoC-1_04_F24), and two from mammals (SmoC-1_02_H05, SmoC-1_03_F21). These data suggest that homologs have either not yet been identified or are absent from other plant lineages, although in one case (SmoC-1_06_O23), a more distantly related *A. thaliana *gene was returned by BLASTX, and in a further three cases, BLASTN analysis of the EST-others database identified potential homologs in *P. patens *(SmoC-1_02_N06, SmoC-1_06_K12) and *S. lepidophylla *(SmoC-1_cn167).

### Highly represented *S. moellendorffii *ESTs

EST copy number can be used to approximate gene expression levels in an organism, although there are artifacts of cDNA library construction that may limit or over-represent certain transcripts [26]. Table 1 summarizes the first 32 most abundantly represented transcripts in the *S. moellendorffii *EST collection, having six or more EST copies in each cluster, with their identities putatively assigned by BLASTX analysis of the assembled contigs. As expected, a large number of the *S. moellendorffii *ESTs are photosynthesis-related genes, with 19 clusters containing 213 ESTs (9% of total sequenced ESTs) corresponding to genes involved in photosynthesis. There were seven clusters matching to core proteins of photosynthesis reaction centers, including four subunits of photosystem I (PSI-G, PSI-H, PSI-L, PSI-N), and three photosystem II proteins (PsbW, OEC23, CP22). There were four contigs corresponding to light-harvesting chlorophyll a/b-binding proteins, including one early light-induced protein. We also found ESTs for the RuBisCO small subunit, carbonic anhydrase, plastocyanin, one subunit of cytochrome *b_6_f *complex, ferredoxin and ferredoxin/NADP oxidoreductase, proteins involved in carbon fixation and photosynthetic electron transport. There were two putative anti-oxidative proteins found within *S. moellendorffii *ESTs: chloroplastic iron superoxide dismutase and catalase, presumably required for the decomposition of superoxide and hydrogen peroxide [27,28]. The BLASTX results show that all of these highly expressed *S. moellendorffii *photosynthetic genes had homologs in *A. thaliana *genome, consistent with previous observation that the photosynthesis machinery has been highly conserved throughout plant evolution.

**Table 1:** The most abundantly represented ESTs in the *S. moellendorffii *cDNA library.

	Cluster	Number of ESTs	Top BLASTX hit in non-redundant protein database		
			
			Accession Number	Best Identity Description	E-value
1	SmoC-1_cn126	105	-	Novel	-
2	SmoC-1_cn125	46	-	Novel	-
3	SmoC-1_cn018	31	SP:P16031	Ribulose bisphosphate carboxylase small subunit) [*Larix laricina*]	8E-51
4	SmoC-1_cn121	25	SP:P04669	Ferredoxin, chloroplast precursor [*Silene latifolia subsp. alba*]	2E-26
5	SmoC-1_cn106	17	PIR:S16294	chlorophyll a/b-binding protein [*Lycopersicon esculentum*]	9E-99
6	SmoC-1_cn107	17	GB:AAM46780	latex plastidic aldolase-like protein [*Hevea brasiliensis*]	1E-164
7	SmoC-1_cn171	17	PIR:S31863	chlorophyll a/b-binding protein [*Pinus sylvestris*]	1E-106
8	SmoC-1_cn011	14	GB:AAC78107	photosystem-1 H subunit GOS5 [*Oryza sativa*]	8E-30
9	SmoC-1_cn233	13	SP:Q9SXW9	Plastocyanin, chloroplast precursor [*Physcomitrella patens*]	2E-37
10	SmoC-1_cn025	11	SP:P51118	glutamine synthetase cytosolic isoenzyme 1 [*Vitis vinifera*]	1E-152
11	SmoC-1_cn089	11	GB:AAG17036	S-adenosylmethionine synthetase [*Pinus contorta*]	7E-17
12	SmoC-1_cn195	11	SP:P11432	Early light-induced protein, chloroplast precursor (ELIP) [*Pisum sativum*]	1E-32
13	SmoC-1_cn023	9	SP:P82977	Subtilisin-chymotrypsin inhibitor [*Triticum aestivum*]	4E-11
14	SmoC-1_cn145	9	-	Novel	-
15	SmoC-1_cn179	9	SP:P30361	Cytochrome B_6_-F complex iron-sulfur subunit 1, chloroplast precursor [*Nicotiana tabacum*]	3E-74
16	SmoC-1_cn189	9	-	Novel	-
17	SmoC-1_cn006	8	GB:AAG59875	PSII subunit PsbW [*Physcomitrella patens*]	5E-13
18	SmoC-1_cn078	8	SP:O48560	Catalase 3 [*Glycine max*]	0
19	SmoC-1_cn211	8	SP:P23993	Photosystem I reaction center subunit XI, chloroplast precursor [*Hordeum vulgare*]	2E-55
20	SmoC-1_cn226	8	PDB:1EKJA	Carbonic Anhydrase [Pisum Sativum]	2E-63
21	SmoC-1_cn019	7	REF:NP_175963	photosystem I reaction center subunit V, chloroplast, [*Arabidopsis thaliana*]	2E-34
22	SmoC-1_cn108	7	PIR:T23512	hypothetical protein K08H10.2a [*Caenorhabditis elegans*]	1E-12
23	SmoC-1_cn215	7	GB:AAB88617	ubiquitin conjugating enzyme [*Zea mays*]	3E-82
24	SmoC-1_cn218	7	SP:P27494	Chlorophyll a-b binding protein 36, chloroplast precursor [*Nicotiana tabacum*]	1E-127
25	SmoC-1_cn013	6	PIR:T06471	core protein [*Pisum sativum*]	1E-20
26	SmoC-1_cn016	6	SP:Q9SLQ8	Oxygen-evolving enhancer protein 2, chloroplast precursor [*Cucumis sativus*]	1E-79
27	SmoC-1_cn033	6	GB:AAM97011	expressed protein [*Arabidopsis thaliana*]	6E-40
28	SmoC-1_cn136	6	GB:AAO49652	photosystem I-N subunit [*Phaseolus vulgaris*]	2E-37
29	SmoC-1_cn139	6	DBJ:BAC66946	chloroplastic iron superoxide dismutase [*Barbula unguiculata*]	3E-69
30	SmoC-1_cn180	6	EMB:CAB71293	chloroplast ferredoxin-NADP+ oxidoreductase precursor [*Capsicum annuum*]	1E-139
31	SmoC-1_cn208	6	SP:P54773	Photosystem II 22 kDa protein, chloroplast precursor [*Lycopersicon esculentum*]	5E-61
32	SmoC-1_cn250	6	-	Novel	-

Non-redundant protein database includes all non-redundant GenBank CDS translations (GB)+ RefSeq Proteins (REF) +PDB + SwissProt (SP) + PIR + PRF. The identities of ESTs were putatively described by the top BLASTX hit (with lowest E-value) of the assembled EST contigs.

Three highly expressed *S. moellendorffii *transcripts corresponded to genes encoding enzymes of metabolism, including an aldolase-like protein, a putative glutamine synthetase cytosolic isoenzyme involved in nitrogen assimilation [29,30], and a putative S-adenosylmethionine synthetase required for the synthesis of the major methyl group donor involved in the methylation of a variety of biomolecules ranging from histones to secondary metabolites, and for the biosynthesis of ethylene [31,32].

Other relatively abundant ESTs included one encoding a putative subtilisin-chymotrypsin inhibitor, exhibiting 49% amino acid sequence identity with the wheat subtilisin-chymotrypsin inhibitor, which may play a role in plant defense by inhibiting the serine proteinases of pathogens [33]. Two transcripts that matched an *A. thaliana *expressed protein and *Pisum sativum *core protein may function as membrane channel proteins. Interestingly, one highly expressed EST matched with an E-value of 10^-12 ^a *C. elegans *protein of unknown function, and is only more distantly related to an *A. thaliana *late embryogenesis abundant protein.

There were five highly expressed ESTs that did not yield significant matches using BLASTX (E>10^-5^). These are putative *Selaginella-*specific genes and may encode proteins with functions unique to *Selaginella *or lycophytes. The first two highly expressed ESTs in this project, represented by clusters SmoC1_cn126 and SmoC1_cn125, had 105 and 46 copies in their clusters respectively, but returned no BLASTX hits with the nr protein database or BLASTN hits with the NCBI EST-others database. To determine whether these sequences represented bona fide *Selaginella *genes, we amplified the corresponding sequences by PCR using genomic DNA as a template (data not shown). Both sequences amplified successfully, and both had introns, indicating that they were not derived from DNA contamination from prokaryotic symbionts. The rational translation of SmoC1_cn126 contig contains a three repeats of the motif "XXXGXXTCDKCAQTGVCTCGKN", which aligns with similar cysteine-rich motifs in proteins with epidermal growth factor repeats. Using a low BLASTX stringency (E = 0.002), SmoC1_cn125 matched to a *Cynodon dactylon *metallothionein-like protein (GB:AAS88721.1, 75% identical within a 20 amino acid motif). The other three highly expressed *S. moellendorffii *specific ESTs lack hints for functional annotation. The biological function of the proteins encoded by these genes, and the question of whether high transcript abundance is predictive of high protein expression will be a matter for future investigation.

### Functional categorization of *S. moellendorffii *ESTs

The most sensitive method to find new members of known gene families among EST sequences is to search for homology of the translated ESTs to motifs extracted from a multiple alignment of known gene family members [18]. To functionally categorize *S. moellendorffii *ESTs using motif homology searches, we translated the 1301 unigenes in six reading frames and imported them into InterProScan [34], which aligned 491 clusters to InterPro entries (E<10^-5^). Mapping of InterPro entries to GO [35], assigned 343 out of 491 InterPro hits with 562 GO accession numbers. The 562 accession numbers further generated 964 individual GO mappings in the three major ontologies (biological processes, molecular functions and cellular components) [36]. The apparent discrepancies between these values arises from the fact that not all InterPro hits had available GO accession numbers associated with them, one InterProScan entry could be assigned to more than one GO accession numbers, and one GO accession number could be mapped under multiple parental categories [37].

**Figure 4:**
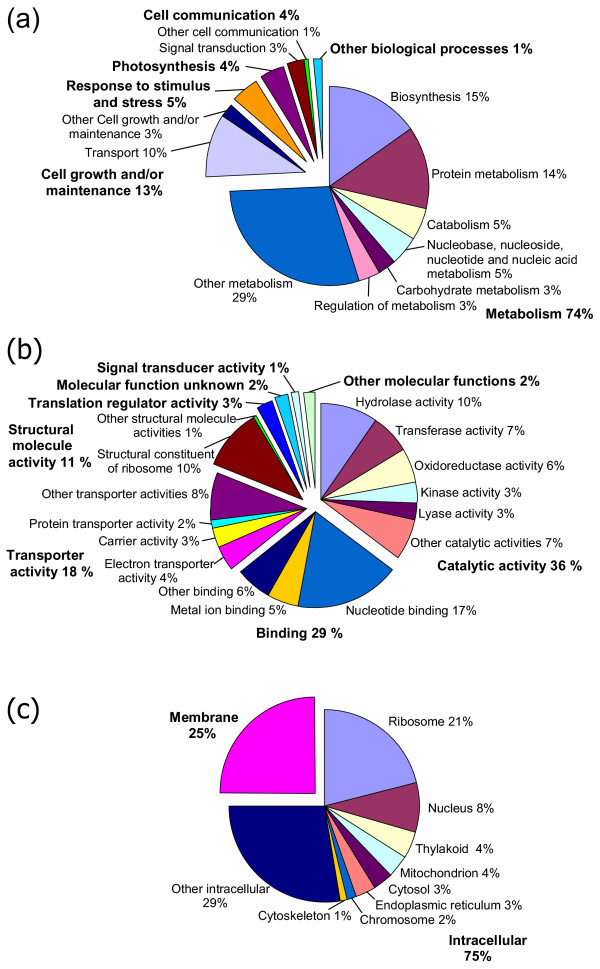
**Representations of Gene Ontology (GO) mapping results for *S. moellendorffii *non-redundant ESTs. **(a) Biological process (b) Molecular function (c) Cellular component.

Tables 2 and Figure 4 summarize the GO assignment of *S. moellendorffii *ESTs in terms of biological processes, molecular functions and cellular components, covering a broad range of the GO functional categories. Using the downloaded *A. thaliana *GO assignments from the TIGR *A. thaliana *Gene Index [38,39], we compared the distribution of GO categories between *S. moellendorffii *ESTs and *A. thaliana *tentative consensus sequences (TCs). Table 3 shows that the distribution patterns of GO assignments of *S. moellendorffii *and *A. thaliana *transcripts were generally similar, with a few exceptions in some categories. Besides the true differences in functional distribution of unigenes, some of the differences could be due to the difference in EST data sources between these two species. For example, in terms of biological processes, *A. thaliana *has a higher percentage in 'response to stimulus and stress' and 'development' than *S. moellendorffii*. Considering that among the *A. thaliana *ESTs in the TIGR database, some were generated from plants at specific developmental stages or from plants exposed to specific biotic or abiotic stimuli, it is very likely that ESTs from orthologs of these genes would be missing from the *S. moellendorffii *ESTs which were generated from normal mature plants.

**Table 2:** The GO categorization of *S. moellendorffii *ESTs by biological process, molecular function, and cellular component.

	Gene Ontology term	Representation	Representation percentage
Biological process	Metabolism	312	74%
	Biosynthesis	64	15%
	Protein metabolism	57	14%
	Catabolism	22	5%
	Nucleic acid metabolism	20	5%
	Cell growth and/or maintenance	53	13%
	Transport	44	10%
	Response to stimulus and stress	19	5%
	Photosynthesis	16	4%
	Cell communication	15	4%
	Signal transduction	12	3%
	Homeostasis	3	1%
	Development	1	<1%
	Cell death	1	<1%
Molecular function	Catalytic activity	132	36%
	Hydrolase activity	36	10%
	Transferase activity	25	7%
	Oxidoreductase activity	22	6%
	Kinase activity	12	3%
	Binding	107	29%
	Nucleotide binding	65	18%
	Metal ion binding	20	5%
	Transporter activity	64	18%
	Electron transporter activity	16	4%
	Carrier activity	12	3%
	Structural molecule activity	40	11%
	Translation regulator activity	10	3%
	Signal transducer activity	4	1%
	Chaperone activity	3	1%
	Enzyme regulator activity	2	1%
	Motor activity	1	<1%
	Transcription regulator activity	1	<1%
Cellular component	Intracellular	135	75%
	Membrane	45	25%

Note that one gene product may be assigned to more than one GO terms, and one children term can fit into multiple parental categories. The representation means the number of non-redundant ESTs that can be mapped to a certain GO term. The representation percentage is based on the total number of GO mappings in each of the three major ontologies (biological process: 420, molecular function: 364, cellular component: 180).

**Table 3:** Comparison of GO assignments between *A. thaliana *ESTs and *S. moellendorffii *ESTs.

Gene Ontology term	Categories	Representation percentage	
		
		*S. moellendorffii*	*A. thaliana*
Biological process	Metabolism	74%	39%
	Cell growth and/or maintenance	13%	13%
	Response to stimulus and stress	5%	16%
	Photosynthesis	4%	<1%
	Cell communication	4%	6%
	Homeostasis	1%	1%
	Development	<1%	6%
	Cell death	<1%	1%
Molecular function	Catalytic activity	36%	41%
	Binding	29%	32%
	Transporter activity	18%	8%
	Structural molecule activity	11%	2%
	Translation regulator activity	3%	1%
	Signal transducer activity	1%	1%
	Chaperone activity	1%	2%
	Enzyme regulator activity	1%	1%
	Motor activity	<1%	1%
	Transcription regulator activity	<1%	7%
Cellular component	Intracellular	75%	70%
	Membrane	25%	19%

The GO assignments for *A. thaliana *ESTs were obtained from TIGR [38]. The percentage of GO assignments for *A. thaliana *was calculated based on the total numbers of GO mappings in each of the three major ontologies with the number of unknown terms deducted from them (biological process: 20185, molecular function: 23680, cellular component: 6309). The functional categories present in *A. thaliana *but not in *S. moellendorffii *were not listed in the table.

The current GO annotations for plants are based solely on the annotated proteins of *A. thaliana *and *O. sativa*, both of which are angiosperms. Since the lycophyte clade diverged from other plant lineages 400 million years ago, and 200 million years before angiosperms, it is perhaps to be expected that a large proportion of *S. moellendorffii *genes could not be accurately assigned to GO categories in the database containing only angiosperm gene entries. We expect that the representation of plant species other than angiosperms will certainly benefit resources as InterPro and in turn will lead to further resolution within GO.

### Comparative genomics of *S. moellendorffii *ESTs

One important objective of comparative genomics is to trace gene evolution including the emergence, development, and loss of orthologous genes in different organisms over evolutionary time [40]. To survey the *S. moellendorffii *ESTs in an evolutionary context, we used the *S. moellendorffii *unigene sequences as queries to search for homologous sequences in the *A. thaliana *and *P. patens *EST databases using tBLASTX algorithm (cut off E-value = 10^-6^). There were two reasons that we chose *A. thaliana *and *P. patens *ESTs as tBLASTX databases. First, *A. thaliana *and *P. patens *are representatives of the most diverged lineages of land plants, namely angiosperms and bryophytes. They flank *Selaginella *in the plant phylogenetic tree, and last shared a common ancestor over 400 million years ago [23], thus providing ample opportunity for the evolutionary divergence of individual genes and gene families. Second, the large quantities of *A. thaliana *and *P. patens *ESTs in GenBank (472,278 and 104,027 respectively) provide a substantial coverage of the transcriptome in these two species. Using them as BLAST databases makes it possible to do a relatively comprehensive genomic analysis even in the absence of the full genome sequence of *P. patens*.

**Figure 5:**
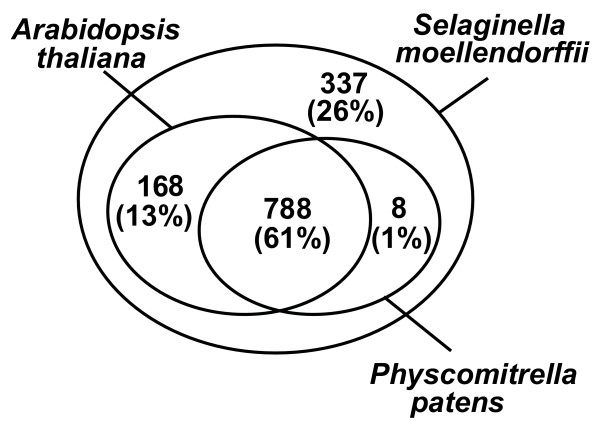
**A Venn diagram showing the distribution of *S. moellendorffii *EST tBLASTX matches by databases.**The 1301 translated *S. moellendorffii *non-redundant ESTs were used as queries in homology searches against *A. thaliana *and *P. patens *EST databases, respectively. The two inner circles contain the numbers and percentages of *S. moellendorffii *ESTs that share tBLASTX similarity with *A. thaliana *or *P. patens *ESTs. The region between inner circles and outer circle represents *S. moellendorffii *ESTs without tBLASTX matches.

Figure 5 summarizes the distribution of *S. moellendorffii *ESTs by tBLASTX results. Among 1301 non-redundant *S. moellendorffii *ESTs, 788 (61%) ESTs had homology with both *A. thaliana *and *P. patens *ESTs. These ESTs probably identify non-dispensable genes, which tend to be evolutionarily conserved in all land plants [41]. 168 (13%) ESTs had exclusive similarity with *A. thaliana *ESTs, and may represent the genes that evolved in land plants after the divergence of bryophytes, or those that were lost from the genomes of mosses. Table 4 shows the top 20 *S. moellendorffii *EST tBLASTX hits for *A. thaliana *ESTs that were not present within the *P. patens *EST database ranked by tBLASTX E-values. Among these, it is possible to identify candidates that might have contributed to the success of vascular plants, including those involved in functions such as lignification (SmoC-1_05_G17) [42], cell division control (SmoC-1_01_E02) [43], intracellular transport (SmoC-1_02_C05 and SmoC-1_05_G03) [44,45], responses to sulfur starvation (SmoC-1_03_C14) [46], dehydration (SmoC-1_06_M11), and viral infection (SmoC-1_06_P21) [47]. Only 8 (1%) *S. moellendorffii *ESTs had similarity only with *P. patens *ESTs. These ESTs may represent genes that arose early in plant evolution but were lost later after the divergence of the lycophytes. It should be noted, however, that all eight of these ESTs had relative low tBLASTX score (E-value around 10^-10^), limiting our certainty that the homologous ESTs in *P. patens *are true orthologs. Finally, there were 337 (26%) ESTs that had no tBLASTX match in the *A. thaliana *and *P. patens *EST databases. These ESTs may be *Selaginella-*specific genes, possibly having evolved only in lycophytes after their divergence from other lineages or having arisen after the divergence of bryophytes and later being lost in euphyllophytes.

## Conclusion

We sequenced 2181 ESTs from the lycophyte *S. moellendorffii*, putatively representing 1301 unigenes. Our data showed that a large proportion of the genes had homologous genes in the well-studied model plant *A. thaliana *and other plant species. By browsing the putative functional annotations of these ESTs, researchers will be able to choose *S. moellendorffii *genes of interest and compare them to their othologs in other species. We also found a substantial number of putative *Selaginella-*specific genes that do not share similarity with known genes, with some of them even representing very highly expressed genes. Considering the complexity of the plant kingdom and a time span more than 150 million years between the divergences of lycophytes and angiosperms, it will not be surprising to identify gene functions in *S. moellendorffii *that are not present in *A. thaliana*. When the draft genome sequence of *S. moellendorffii *is completed and released, this EST resource will also play an important role in the mapping and annotation of the genome. As a member of a clade that arose after the bryophytes and before all other vascular plants, *S. moellendorffii *will provide new opportunities in studying plant evolution, particularly those adaptations relating to fundamental traits that facilitated the transition of green plants to the land, such as lignification in vascular plants, root/stem/leaf organography, complex patterns of sporophyte branching, and the elaboration of reproductive structures.

## Methods

### Plant material and cDNA library Construction

*S. moellendorffii *was obtained from Plant Delights Nursery (Raleigh, NC). Plants were grown at 23°C in a greenhouse with a photoperiod of 16h light/8h dark. The cDNA library used in this study was made from RNA extracted from pooled tissue including stems, microphylls, strobilis, and rhizophores of *S. moellendorffii *plants. Briefly, fresh tissue was ground in liquid nitrogen and total RNA was extracted using the RNeasy Max Kit (QIAGEN, Valencia, CA), treated with RNase-free DNase, and precipitated in 2 M lithium chloride. Poly A+ RNA was isolated from total RNA using the Dynabeads mRNA Purification Kit (Dynal Biotech, Brown Deer, WI). The cDNA library was constructed from 1 μg mRNA using the Creator Smart cDNA Library Construction Kit (CLONTECH, Palo Alto, CA). After first-strand synthesis, the full length double stranded cDNAs were synthesized by primer-extension. Full length double stranded cDNAs were digested with *Sfi *I and size fractionated using a CHROMA SPIN-400 column (CLONTECH, Palo Alto, CA). cDNA-containing fractions were pooled, and ethanol precipitated. The cDNAs were then cloned into pDNR-LIB at *Sfi *I site, and electroporated into *E. coli *DH10B cells (Invitrogen, Carlsbad, CA). The library had an un-amplified titer of 1.6 × 10^6 ^colony-forming units mL^-1 ^and a total complexity of 3.2 × 10^6 ^colonies. To estimate the average insert size of the library, plasmid DNAs were extracted from 35 randomly picked clones from the library, digested with *Sfi *I, and analyzed by agarose gel electrophoresis.

### EST sequencing and dbEST submission

18,432 colonies from un-amplified *S. moellendorffii *cDNA library were arrayed into 48 384-well plates using Q-Pix multifunction colony picker (Genetix). Plasmid DNA was isolated from 2304 clones picked from the first six 384-well plates. Sequences of cDNAs were determined from their 5' end by conventional procedures using the big-dye terminators on the ABI 3730xl DNA analyzer (Applied Biosystems, Foster City, CA) at the Purdue Genomics Center using T7-ZL (5'-TAATACGACTCACTATAGGG-3') as the 5'-sequencing primer. The vector sequence was trimmed from the original EST sequences resulting in 2181 sequences. The 2181 ESTs have been submitted to GenBank dbEST under the accession numbers DN837577 to DN839757 [20].

### EST clustering and homology search

2181 EST sequences were imported into the stackPACK v2.2 clustering system (Electric Genetics, Reston, VA) through WebPipe for clustering with default setting, and contig consensus sequences were generated from the clusters. One thousand three hundred and one non-redundant EST sequences were exported through WebReport in FASTA format. BLASTX analyses using the nr database were performed on the 1301 unigene sequences, using E-value of 10^-5 ^as a cutoff threshold. The complete BLASTX annotation of 1301 *S. moellendorffii *unigenes can be viewed at [48].

### Functional categorization of ESTs

To search for functional protein domains of translated ESTs, 1301 unigene sequences were merged into one FASTA file and imported into InterProScan, which was run on a local SUN unix server. BlastProDom, Coil, FPrintScan, HMMPIR, HMMPfam, HMMSmart, HMMTigr, ProfileScan, ScanRegExp, and Seg superfamily were selected as the database methods. All the sequences were translated in six reading frames and aligned to the entries in the selected databases. EST clusters which had positive InterProScan hits (E <10^-5^) were automatically assigned InterPro accession numbers. According to the mapping of InterPro entries to GO [35], GO accession numbers were assigned to EST clusters, which were used to classify ESTs into functional groups by molecular function, cellular component, and biological process. In comparison of the distribution of GO categories between *S. moellendorffii *ESTs and *A. thaliana *TCs, the GO assignments for A. thaliana ESTs were obtained from TIGR [38]. The Complete Interpro assignment and GO mapping of *S. moellendorffii *ESTs can be accessed in the supplemental data (see Additional file: 1). 

### Comparison of *S. moellendorffii *ESTs to *A. thaliana *and *P. patens *ESTs

472,278 *A. thaliana *ESTs and 104,027 *P. patens *ESTs retrieved from GenBank by searching '*Arabidopsis / Physcomitrella *and gbdiv est' in NCBI Entrez [25] were saved to a local server. The 1301 *S. moellendorffii *unigenes were translated in six reading frames and searched for homology against the six-frame translations of *A. thaliana *ESTs and *P. patens *ESTs respectively using the BLAST algorithm. An E-value of 10^-6 ^was set as stringency threshold. The complete result of *S. moellendorffii *unigenes tBLASTX against *A. thaliana *and *P. patens *ESTs can be viewed at [48].

### Genomic PCR

To amplify the genomic sequences of the two most highly expressed ESTs (SmoC1_cn126 and SmoC1_cn125) in *S. moellendorffii*, PCR was performed using genomic DNA extracted from 50 mg fresh tissue of *S. moellendorffii *as described previously [49] as template and two pairs of PCR primers designed from their EST contig sequences: CC1170 (5'-CGAGCTCGTAGTGATAGTGTC -3') and CC1171 (5'-AACCATAGGAGAGGAAGACC-3') for SmoC1_cn126; CC1228 (5'-ATAGCTTAGCTGCTTTCTTCTC-3') and CC1229 (5'-ATACTACTCATGTCGCAGCTC -3') for SmoC1_cn125. PCR was performed using an initial 2 min denaturation at 94°C, followed by 25 cycles, each consisting of a 0.5 min denaturation at 94°C, a 0.5 min annealing at 50°C, and a 1 min extension at 72°C. These 25 cycles were followed by a 5 min extension at 72°C. PCR products were purified using QIAquick PCR Purification Kit (QIAGEN) and sequenced at Purdue Genomics Center.

## Authors' contributions

JKW constructed the *S. moellendorffii *cDNA library, participated in the EST sequencing, carried out the bioinfomatic analysis of the ESTs, and performed the genomic PCR for two transcripts. MT participated in the *S. moellendorffii *cDNA library construction and provided comments on the manuscript. CC conceived the study and coordinated work. JKW and CC wrote the article. All authors read and approved the final manuscript.

**Table 4:** Top 20 *S. moellendorffii *EST tBLASTX hits for *A. thaliana *ESTs that are not present within the *P. patens *EST database.

	Non-redundant EST	tBLASTX E-value	Best BLASTX Descriptor in *A. thaliana*	Accession Number
1	SmoC-1_01_H05	1E-107	expressed protein	REF:NP_194688
2	SmoC-1_02_C05	1E-99	oligopeptide transporter OPT family protein	REF:NP_192815
3	SmoC-1_01_L23	2E-99	putative Mg-protoporphyrin IX chelatase	REF:NP_196867
4	SmoC-1_05_G17	2E-99	putative caffeoyl-CoA 3-O-methyltransferase	REF:NP_195131
5	SmoC-1_05_K13	5E-90	chloroplast membrane protein (ALBINO3)	REF:NP_180446
6	SmoC-1_01_E02	1E-89	cullin family protein	REF:NP_567243
7	SmoC-1_05_G03	7E-87	putative UDP-galactose/UDP-glucose transporter	REF:NP_563949
8	SmoC-1_05_I19	6E-86	expressed protein	REF:NP_566060
9	SmoC-1_02_N15	9E-86	nicotinate phosphoribosyltransferase family protein	REF:NP_179923
10	SmoC-1_03_I01	7E-80	glycoside hydrolase family 77 protein	REF:NP_181616
11	SmoC-1_cn293	4E-77	amine oxidase family protein	REF:NP_181830
12	SmoC-1_03_C24	9E-73	uridylyltransferase-related protein	REF:NP_564010
13	SmoC-1_02_P14	5E-70	expressed protein	REF:NP_199542
14	SmoC-1_06_P21	2E-69	RNase L inhibitor protein-related	REF:NP_196569
15	SmoC-1_05_G10	3E-69	expressed protein	REF:NP_191746
16	SmoC-1_03_C14	1E-66	putative isoflavone reductase	REF:NP_565107
17	SmoC-1_03_N06	6E-65	transducin / WD-40 repeat family protein	REF:NP_190148
18	SmoC-1_06_M11	1E-63	dehydration stress-induced protein	GB:AAM62648
19	SmoC-1_06_B20	1E-60	putative membrane protein	REF:NP_849987
20	SmoC-1_05_O21	2E-60	paired amphipathic helix repeat-containing protein	REF:NP_186781

The tBLASTX E-value of an EST varies with its BLASTX E-value in a small range (e.g. SmoC-1_01_H05 has a tBLASTX E-value of 1E-107 against its homologous *A. thaliana *EST and a BLASTX E-value of 2E-94 against the translated full length *A. thaliana *cDNA.). The homology ranking was based on the tBLASTX E-value. The identities of ESTs were putatively described by the *A. thaliana *protein with the lowest E-value in the BLASTX analysis.

## Supplementary Material

Additional file 1 The Complete Interpro assignment and GO mapping of S. moellendorffii ESTs, Excel file. Click here for file
